# Effects of Low-dose Morphine on Nitric Oxide Concentration and Angiogenesis in Two-kidney One Clip Hypertensive Rats

**Published:** 2011

**Authors:** Aliasghar Pourshanazari, Mohammad Allahtavakoli, GHGholamhossein Hassanshahi

**Affiliations:** 1 *Physiology and Pharmacology Research Centre, Rafsanjan University of Medical Sciences, Rafsanjan, Iran*; 2 *Molecular Medicine Research Centre, Rafsanjan University of Medical Sciences, Rafsanjan, Iran*

**Keywords:** Angiogenesis, Blood pressure, Morphine, Nitric oxide, Renin activity

## Abstract

**Objective(s):**

We investigated the effects of low-dose morphine on nitric oxide (NO) and angiogenesis in two-kidney one clip hypertensive (2K1C) rats.

**Materials and Methods:**

Male rats were divided into two groups: sham-clip operated and 2K1C. Each group subdivided into saline and morphine (3 mg/kg i.p. 8 weeks) groups. Blood pressure, heart rate, plasma renin activity (PRA), NO concentration and murine matrigel angiogenesis were evaluated.

**Results:**

Morphine had no effects on blood pressures and HR in sham normotensive rats but attenuated diastolic blood pressure (DBP) (*P*< 0.01) and mean arterial pressure (MAP) (*P*< 0.01) in 2K1C compared with saline. PRA level was significantly higher in 2K1C compared with sham groups (*P*< 0.01) but morphine decreased it in 2K1C compared with saline (*P*< 0.01). After clipping, NO in 2K1C hypertensive rats was decreased (*P*< 0.01) and morphine increased it compared with saline (*P*< 0.01). Morphine promoted angiogenesis in both sham (*P*< 0.01) and 2K1C (*P*< 0.0001) groups.

**Conclusion:**

Low-dose morphine stimulated angiogenesis in two-kidney one clip hypertensive rats probably via NO pathways.

## Introduction

Hypertensive patients are at risk for cardiovascular complications related to endothelial dysfunction and angiogenesis (1). Angiogenesis is the process of formation of new vasculature and expansion of the capillary network from preexisting vessels which is essential for regeneration and tissue repair (2). Vascular endothelial function is well correlated with angiogenesis and the endothelium promotes vasodilation, inflammation and vascular smooth cell proliferation by releasing nitric oxide (NO) (3). NO as a biological messenger is known to be involved in diverse physiological and pathophysiological processes in various organ systems. NO is a potential regulator for angiogenesis and many studies have shown that NO is closely involved in the regulation of systemic blood pressure (4). 

In the other hand, morphine is commonly used to control severe pain and also in addicted persons. Whereas the mechanisms of opioids have been mostly characterized in nervous system, little is known about their systemic effects. Some studies have shown the presence of specific opioid receptors, including MOR on the endothelial cells (5,6). Moreover, morphine induces NO in the endothelium and some other tissues and leads to vasodilation (7). Opioids also promote cell proliferation in non endothelial cells (9,10). These findings show NO- dependent angiogenesis is induced by low-dose of morphine, but there are some evidence which shows that endothelial cells express a local circuit regulatory pathway driven by endogenous opiates and constitutive (11). However, very few studies are available investigating the effect of morphine on angiogenesis in hypertensive animals and results are controversial (12-15). For example, high-dose morphine has cytotoxic effects in macrophages (16), vascular endothelia (17), mesangial, and epithelial cells (18).

Because the effects of morphine on these processed remains unknown, we examined the effects of prolonged low-dose morphine on induction of hypertension and angiogenesis in two-kidneys one clip reno vascular hypertensive (2K1C) rats.

## Materials and Methods

Thirty two male Wistar rats with initial weight of 200±20 gram were enrolled in this study and housed at a controlled temperature with free access to food and water. The animals were randomly divided into 2 groups: sham normotensive and 2K1C hypertensive rats. Each group was divided into saline and morphine receiving groups (n= 8). Eight weeks immediately after 2K1C surgery 3 mg/kg of Morphine sulphate was injected i.p. and the saline group received the same volume of saline in according to a similar protocol. Surgeries and protocols were performed as follow:

***Preparation of hypertensive rats by 2K1C gold blatt method***

The rats were anesthetized with ketamine hydrochloride (60 mg/kg) and xylazine (7.5 mg/kg) intraperitonealy. Left kidneys were exposed via flank incision and a silver clip with internal gap of 0.2 mm was put around the renal artery. In the sham group, the same procedure was done without using silver clip. Penicillin G (25000 U IM) was injected after surgery. Rats were fed a commercial rat chow (Razi Institute, Iran) and had free access to tap water. A few days after placement of the clip, the systolic blood pressure (SBP) was measured twice a week with the tail-cuff method (AD instrument Australia). After 8 weeks, the animals were anesthetized and direct blood pressure was measured by a catheter (PE50) inserted into femoral artery. Blood samples were taken for subsequent determination of plasma renin activity (PRA).

***Plasma renin activity assay***

PRA was measured with a kit from Diasorin Inc. using ^125^-I Angiotensin I generation. Angiotensin I coated-tube radioimmunoassay (RIA) was performed in two aliquots of the same sample, one incubated at 37 ºC for generation and one non-incubated; PRA was calculated as ng angiotensin I generated/ml/h (Renctk P2721, Sorin-Biomedica Diagnostic Division RIA kit, Italy). The PRA assay sensitivity was 0.13 ng/ml; intra-and interassay coefficients of variation were 7.5 and 7.7%, respectively.

***Protocol for determining serum nitric oxide concentration***

From all animals blood samples were taken before and after the study. Serum NO concentration was measured by Gris reagent system (Promega Corporation, Madison, USA) and using available reagents. Serums were added into wells (96-well flat-button enzymatic assay plate). A sulfanilamide solution was added to all collected samples and then *N*-1-naphtylethylenediamine dihydrochloride (NED) was added under acidic conditions. The absorbances were detected in 520-550 nm wavelengths by a microreader (19). NO concentration in the samples was determined by comparison to nitrite standard curve. The limitation of detection was 2.5 µM nitrite.

***Murine matrigel angiogenesis assay***

Angiogenesis was assessed *in vivo* using 500 μl of Matrigel (BD Bioscience, San Jose, CA) containing fibroblast growth factor (10 ng/ml, R&D System, Minneapolis, MN) and heparin (60 U/ml, Braun Melsungen AG, Melsungen, Germany) which was injected subcutaneously into the abdominal wall of rats 8 weeks after the commencement of morphine or saline administration (as described earlier). Ten days later, 25 mg/ml FITC-dextran was injected systemically, and blood samples were collected. After sacrificing the rats, Matrigel implants were excised and photographed under a fluorescent microscope and then homogenized with 5 units/ml dispase (Life Technologies, Inc., Grand Island, NY). Angiogenic response was expressed as the fluorescence ratio of Matrigel implant: plasma, obtained using a Fluorescence Multi Plate Reader (Applied Biosystems, Foster City, CA) (20). 

***Statistical analysis***

The results are presented as Mean±SEM. Data were compared by an unpaired t-test or ANOVA and Tukey as a post test as appropriate. Statistical significance was accepted at a level of *P*< 0.05.

## Results

***Blood pressure and heart rate***

**Figure 1.**
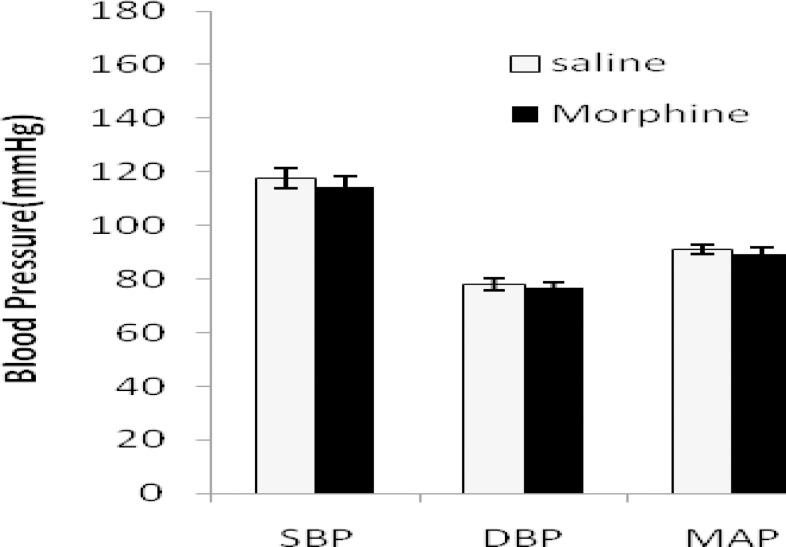
Systolic blood pressure (SBP), diastolic BP (DBP) and mean arterial pressure (MAP) in sham-clip operated normotensive rats in morphine (3 mg/kg.i.p./day 8 weeks after surgery) and saline receiving groups. Values are mean±SEM. Data were analyzed by unpaired t-test and no statically significant difference was found between the saline and morphine groups (n= 8).

**Figure 2.**
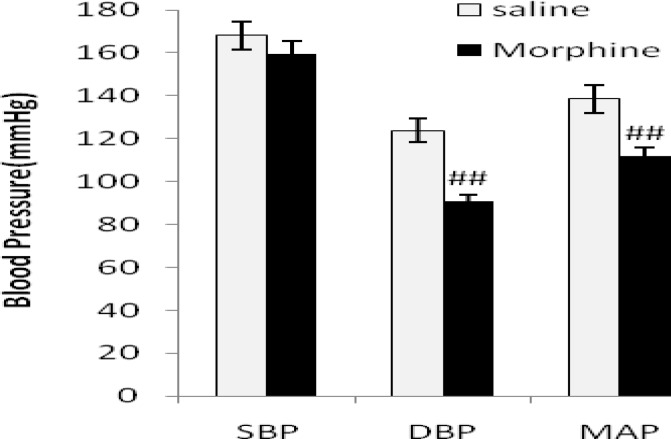
Systolic blood pressure (SBP), diastolic BP (DBP) and mean arterial pressure (MAP) in 2K1C hypertensive rats in morphine (3 mg/kg.i.p./day 8 weeks after surgery) and saline receiving groups. Values are mean±SEM. Data were analyzed by unpaired t-test and statically significances were shown between the saline and morphine groups (*P*< 0.01, n=8).

**Figure 3.**
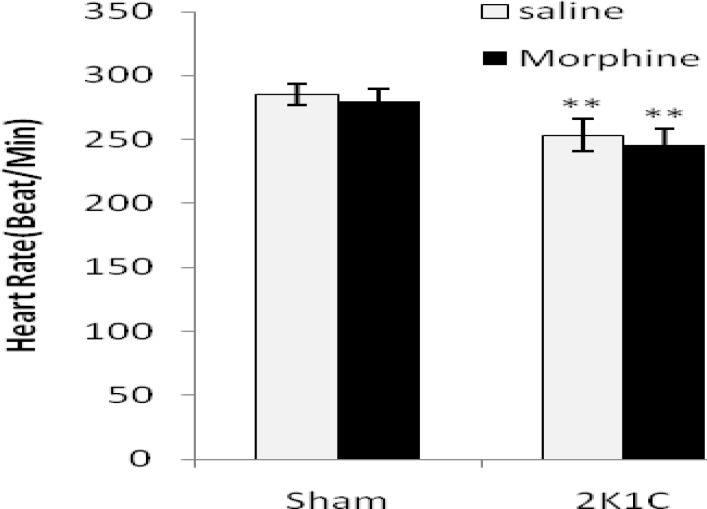
Heart rate (beats/min) in sham-clip operated normotensive and 2K1C hypertensive rats in morphine (3 mg/kg. i.p./day 8 weeks after surgery) and saline receiving groups. Values are mean±SEM. Data were analyzed by ANOVA and statically significances were shown between the sham and 2K1C groups (**P< 0.01). Statistical analyses were done within two hypertensive groups by unpaired t-test and no significant differences were found between saline and morphine groups (n= 8).

Systolic blood pressure (SBP), diastolic blood pressure (DBP), mean arterial pressure (MAP), and heart rate (HR) are shown in Figure 1-3 in all groups. In 2K1C group SBP and DBP increased significantly (*P*< 0.01) and HR decreased (*P*< 0.01) compared with sham animals. Morphine had no effects on blood pressures and HR in sham normotensive rats but attenuates DBP (*P*< 0.01) and MAP (*P*< 0.01) in 2K1C compared with saline receiving group (Figure1 to 3).

***Plasma renin activity***

**Figure 4.**
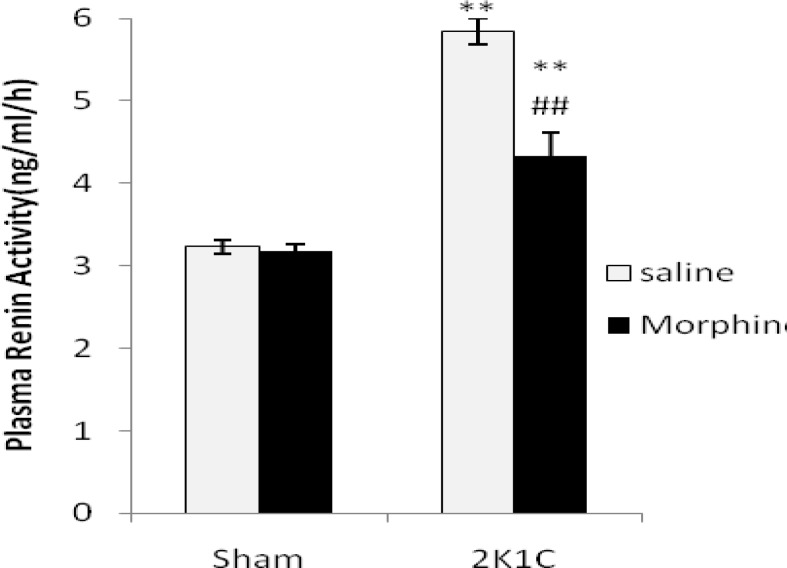
Plasma renin activity (PRA) values (ng/ml/h) in sham-clip operated normotensive and 2K1C hypertensive rats in morphine (3 mg/kg. i.p./day 8 weeks after surgery) and saline receiving groups. Values are mean±SEM. Data were analyzed by ANOVA and statically significant differences were shown between the sham and 2K1C groups (**P< 0.01). Statistical analyses were done within two hypertensive groups by unpaired t-test and significant differences were shown between saline and morphine in 2K1C groups (*P*< 0.01, n= 8).

Plasma renin activity (PRA) level was significantly higher in 2K1C (*P*< 0.01) in comparison with sham groups (Figure 4). Morphine decreased PRA in 2K1C compared with saline receiving group (*P*< 0.01) but PRA in 2K1C morphine group was still higher than Sham (*P*< 0.01)

***Serum NO concentration***

**Figure 5.**
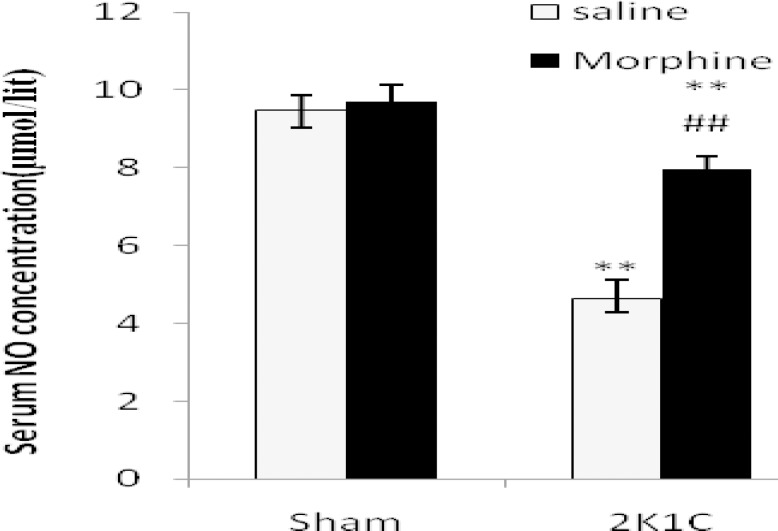
Serum NO concentration (µmol/lit) in sham-clip operated normotensive and 2K1C hypertensive rats in morphine (3 mg/kg. i.p./day 8 weeks after surgery) and saline receiving groups. Values are mean±SEM. Data were analyzed by ANOVA and statistically significant differences were shown between the sham and 2K1C groups (**P< 0.01). Statistical analyses were done within two hypertensive groups by unpaired t-test and significant differences were shown between saline and morphine groups (* P*< 0.01, n= 8).

Figure 5 illustrates serum NO concentration in normotensive sham and 2K1C hypertensive rats. Morphine had no effect on serum NO concentration in sham-clip operated animals (*P*> 0.05). After clipping, serum NO concentration in 2K1C hypertensive rats was decreased and it was significantly lower than sham-clipped group (*P*< 0.01). Morphine improved serum NO level in 2K1C group (*P*< 0.01) but NO was still lower than 2K1C-saline group (*P*< 0.0001).

***Effects of morphine in angiogenesis***

**Figure 6.**
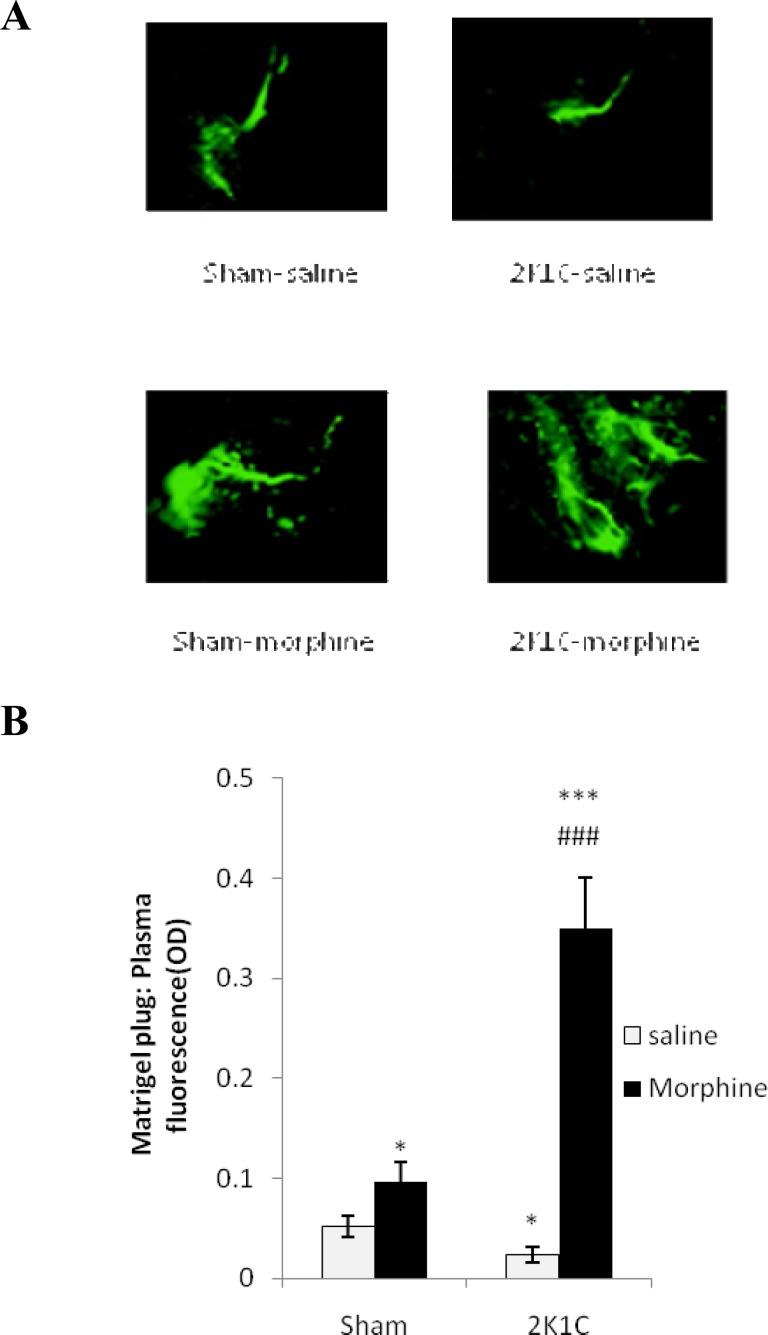
Angiogenesis assay in Matrigel plugs implanted subcutaneously into the rat sham-clip operated normotensive and 2K1C hypertensive rats in morphine (3 mg/kg. i.p./day 8 weeks after surgery) and saline receiving groups. The green fluorescence secondary antibody was detected by a laser scanning confocal imaging system (10×). Low- dose morphine improved angiogenesis on the Matrigel plugs in sham–clip operated groups compared with saline. Data were analyzed by ANOVA and statistically significant difference were shown between the sham and 2K1C groups (*P< 0.05, **P< 0.0001). Statistical analysis were done within two hypertensive groups by unpaired t-test and significant differences were shown between saline and morphine groups (*P*< 0.0001). 2K1C hypertension significantly impairs Angiogenesis compared with sham operated rats (*P*< 0.01). Morphine promoted angiogenesis in 2K1C group in comparison with saline (*P*< 0.0001, n= 8).

Low-dose morphine stimulated angiogenesis on the Matrigel plugs in sham–clip operated groups compared with saline (Figure 6). 2K1C hypertension significantly impairs angiogenesis compared with sham operated rats (*P*< 0.01) but morphine promoted angiogenesis in both sham-clip (*P*< 0.01) and 2K1C (*P*< 0.0001) groups in comparison with saline. 

## Discussion

Our results showed that prolonged low dose morphine did not change blood pressure and heart rate in sham-clipped animals. Blood pressure and PRA increased and heart rate decreased in 2K1C compared with sham-clipped group. Prolonged low dose morphine prevented the promoting of PRA and hypertension in 2K1C compared with saline. Serum NO concentration was significantly decreased after clipping in 2K1C saline receiving group but morphine induced NO in 2K1C group compared with saline.

Studies indicated that in early phase of 2K1C hypertensive animals, increased activity of PRA and renin-angiotensin-aldosterone is responsible for increasing blood pressure (21,22), however, after 8 week of clipping, change of endothelial function is importance regarding hypertension. Reduced blood pressure after prolonged use of low-dose morphine in 2K1C animals may be due to decreased PRA level (23).

We also found that serum NO level was reduced in hypertensive group but prolonged use of low-dose morphine reversed blood pressure, PRA and NO close to normotensive levels. Abnormality in endothelium function which is characterized by decreased in NO concentration, is an important risk factor in hypertension. Endothelial dysfunction in releasing endothelium-derived relaxation factors such as NO and impaired endothelium–dependent relaxation have been demonstrated in several animal models of hypertension and clinical studies (24-27). It suggests that abnormality of NO pathway in hypertension may be due to lower NO production and/or higher NO degradation. Moreover, an increase in reactive oxygen species generation and lower level of antioxidants were detected in hypertensive subjects (28). In addition, lower NO production may be due to reduced L-argenine or endothelium nitric oxide synthase expression in hypertensive rats (29). Thus, reduced serum NO concentration in 2K1C hypertensive rats may be due to cardiovascular effects of hypertension.

Furthermore, 2K1C model induces renin-angiotensin-aldosterone dependent hypertension and another explanation for reduced NO bioavailability in this model of hypertension is that, high angiotensin II level decreases NO level by promoting oxidative stress (30). We also found that prolonged low dose morphine reversed blood pressure and serum NO concentration close to normal level. 

A predominantly antihypertensive role has been reported for endogenous morphine- NO signaling events. Therefore morphine modulates endothelial function and vascular endothelial cells functionality and expresses a local paracrine-autocrine regulatory pathway by endogenously expressed authentic morphine, and constitutive NO. Moreover, considerable evidence shows that NO signaling pathway plays essential role in opioid receptor-mediated responses in the neurocardiovascular system (31-33).

We also examined the effect of low-dose morphine on angiogenesis using *in vivo* Matrigel assays. Our result shows that 2K1C hypertension impaired angiogenesis and low-dose morphine promote it. Some studies showed that hypertension is associated with several vascular abnormalities including vascular rarefaction and endothelial dysfunction (34-36). 

The literatures are mixed concerning the mechanism of morphine effects on angiogenesis. Gupta *et al* reported that low dose morphine stimulates angiogenesis especially in tumor (37). They showed that morphine acts via NO to induce cell proliferation and they believe that morphine signaling and angiogenesis are similar to vascular endothelial growth factor (VEGF). Other studies have shown that the morphine induced mitogenic and survival signaling is comparable with the effect of VEGF on the endothelium (38-40).

Changes of angiogenesis during consumption of morphine have been demonstrated in several experimental studies and the results are controversial. Some studies have shown that high-dose morphine reduces blood vessel proliferation (41) and increased production of superoxide anions in endothelial cells (42). Roy *et al* demonstrated that morphine inhibits VEGF during hypoxic condition in a dose-dependent fashion (43). Chen-Fuh Lam *et al* demonstrated that high-dose morphine was associated with impaired angiogenesis and mobilization of progenitor endothelial cells (12). We suggest that morphine is cytotoxic to endothelial cells at high concentrations. Therefore, the inhibition of angiogenesis, which observer by others may be due to high concentration of morphine and its cytotoxic effect.

## Conclusion

Previously, little was known about the effect of low-dose morphine on the blood pressure and angiogenesis in hypertensive subjects. Based on our data, low-dose morphine prevents induction of hypertension and stimulates angiogenesis in two-kidney one clip hypertensive rats probably via NO pathways. The cardioprotective and proangiogenic activities of low-dose morphine that are shown here might have implications for its therapeutic application in cardiovascular medicine.
